# Evaluation of quantitative parameters of dynamic contrast-enhanced magnetic resonance imaging in qualitative diagnosis of hepatic masses

**DOI:** 10.1186/s12880-018-0299-8

**Published:** 2018-12-27

**Authors:** Jingjing Chen, Youjiao Si, Kaikai Zhao, Xianglong Shi, Weiqun Bi, Shi-en Liu, Hui Hua

**Affiliations:** 1 Department of Radiology, The Affiliated Hospital of Qingdao University, Qingdao, 266000 Shandong Province China; 2 Department of Radiology, Yantai Affiliated Hospital of Binzhou Medical University, Yantai, 264100 Shandong Province China; 3 Department of Oncology, Yantai Affiliated Hospital of Binzhou Medical University, Yantai, 264100 Shandong Province China; 4 Department of Thyroid Surgery, The Affiliated Hospital of Qingdao University, No. 16 Jiangsu Road, Qingdao, 266000 Shandong Province China

**Keywords:** Hepatic tumor, Magnetic resonance imaging, Hemangioma, Hepatocellular carcinoma, Cholangiocarcinoma, Metastatic tumor

## Abstract

**Background:**

To explore the value of parameters of multiphase dynamic contrast-enhanced magnetic resonance imaging (MDCE-MRI) in the qualitative diagnosis of hepatic masses.

**Methods:**

Eighty patients with hepatic masses were retrospectively analyzed. All the patients underwent MDCE-MRI at 3.0 T MR before treatment. Mean enhancement time (MET), positive enhancement integral (PEI), a maximum slope of increase (MSI), and a maximum slope of decrease (MSD) were measured.

**Results:**

There were significant differences between benign and malignant hepatic masses with respect to MET, PEI, and MSI values. The PEI and MSI values between hemangiomas, hepatocellular carcinomas (HCCs), cholangiocarcinomas, and metastatic tumors had significant differences. The MSD value between metastatic tumors, HCCs, and hemangiomas were significantly different. The area under the curve (AUC) values of the receiver operator characteristic curves for MET, PEI, and MSI were 0.70, 0.72, and 0.80, respectively. The specificity of MET, PEI, and MSI were all 77%, and the sensitivities of MSI was the highest, of which was 82.40%. Logistic regression analysis showed the regression equation to be *P* = 1/[1 + e^0.008 × 1 + 0.007 × 2–6.707^], and taking the Youden index maximum points as a diagnostic point was 0.2946.

**Conclusion:**

Some parameters of MDCE-MRI have significant roles in differentiating hepatic masses.

## Background

There are numerous causes of solid hepatic masses, both benign and malignant. It is important to make a correct diagnosis, especially when the potential for therapy exists. While most of these lesions present as solitary masses, multiple lesions may be seen in patients with hemangiomas, hepatocellular carcinomas (HCCs), cholangiocarcinomas, and metastatic tumors. The detectability of hepatic tumors is rising in China with advances in detection of tumor biomarkers and imaging technology [1]. However, the positive predictive value of alpha-fetoprotein (AFP) in the diagnosis of primary liver cancer is only 67.8–74.4% [2]. Although biopsy and histopathologic verification are mandatory for the definitive diagnosis of hepatic masses, those cannot be regarded as ideal diagnostic methods as invasive procedures.

Recently, an increasing interest has been preferred to diagnosing hepatic masses non-invasively. Although magnetic resonance imaging (MRI) has high soft tissue resolution, its routine sequences are not satisfied in the diagnosis of hepatic masses which have the similar imaging appearances on both T1WI and T2WI in most hepatic masses [3–6]. In the last decade, molecular and functional methods of MRI, such as dynamic contrast-enhanced MRI, diffusion-weighted MRI, perfusion-weighted MRI, diffusion tensor imaging, and MR spectroscopy have been investigated to improve the diagnostic capability of MRI.

Dynamic-enhanced CT and MR scanning technology can effectively reflect tumor neovascularization, But MR soft tissue resolution is much higher than that of CT. The organizational structure and hemodynamics are different in different hepatic lesions, resulting in varying dynamic contrast-enhanced MRI manifestations. So, MRI will play more important roles in the qualitative diagnosis of hepatic masses, especially on multiphase dynamic contrast-enhanced MRI (MDCE-MRI) [7].

Liver acceleration volume acquisition (LAVA) is a fast 3D volumetric T1-weighted fat-suppressed imaging technology [8], which depends on the design idea of GE high-density target coils that improve the acquisition speed by parallel acquisition techniques and achieving higher time resolution with the premise of ensuring enough spatial resolution within and between the levels. LAVA automatically uses K space-filling technology and piecewise special technology, which greatly shortens the scan time and obtains good fat suppression. Compared to the previous abdominal multi-period dynamic-enhanced scanning technology, the speed and resolution of LAVA can be improved by 25%, and the scanning range can also be increased by 25%. Clearly, LAVA improves the ability to display minimal change, and is widely used in abdominal lesions. The fine structure of liver lesions can be shown by LAVA involving the different morphology, growth mode, and dynamic enhanced features. Meanwhile, LAVA can also help to make a differential diagnosis of various pathologic types of tumors.

In this present study, we applied LAVA technology to eighty patients with hepatic masses to perform multiphase dynamic contrast-enhanced examinations in an effort to explore the diagnostic value of some quantitative and semi-quantitative parameters of MDCE-MRI in hepatic masses, and to provide a theoretical basis for the qualitative diagnosis of hepatic masses using non-invasive MRI.

## Methods

### Patient population and grouping

**Fig. 1:**
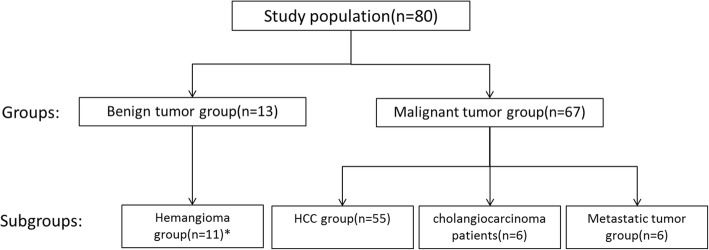
Flow diagram of enrolled patients. *There were only two patients suffering from focal nodular hyperplasia, which was not included in statistics

Between 2011 and 2014, a total of 80 patients (62 males and 18 females) with pathologically-confirmed hepatic masses by surgery or biopsy were scanned by MRI prior to surgery or biopsy. The mean age of the patients was 54 years (range, 26~78 years). The MR examination and diagnostic time interval were all within 30 days. None of the patients received anti-tumor therapy before MR examination and surgery. Informed consent was obtained from each patient prior to the MRI examination. The study was approved by the local ethics committee. Eighty cases were divided into 2 groups (benign and malignant tumor) according to pathologic results, and then, subdivided into 4 groups (hemangioma, HCC, cholangiocarcinoma, and metastatic tumor groups) further. Only two patients were suffering from focal nodular hyperplasia, which was not included in statistics (Fig. 1).

### MRI

All the examinations were performed in GE Signa HDx 3.0 T clinical MRI system using an eight-channel phased array body coil. Gadolinium-diethylene triamine pentaacetic acid (Gd-DTPA) acted as a contrast agent in a concentration of 0.5 mmol/ml. All of the patients fasted for 12 h before the examination in the morning. During the examination the patients were asked to maintain eupnea and avoid abdominal breathing. The following MRI sequences were obtained: (1) axial fast spin echo (FSE) T2-weighted imaging (T2WI) sequence: Repetition Time (TR) 8000 ms, Echo Time (TE) 90 ms, slice thickness 6.0 mm, slice gap 2.0 mm, Matrix 256 × 192. (2) axial fast spin echo (FSE) T1-weighted imaging (T1WI) sequence: TR 150 ms, TE 2.0 ms slice thickness 6.0 mm, slice gap 2.0 mm, Matrix 256 × 192. (3) Multiphase dynamic contrast enhanced MRI: Applying liver acquisition with volume acceleration (LAVA) technologic, TE 1.16 ms; TR 2.52 ms; slice thickness 5.0 mm, slice gap 0 mm, scanning time 120 s, Matrix256x192. During contrast scan, a high-pressure injector was used at a dose of 0.2 mmol/kg of body weight and an injection rate of 3 ~ 4 ml/s following by 20 ml of physiologic saline in the same velocity.

### Images analysis

**Fig. 2:**
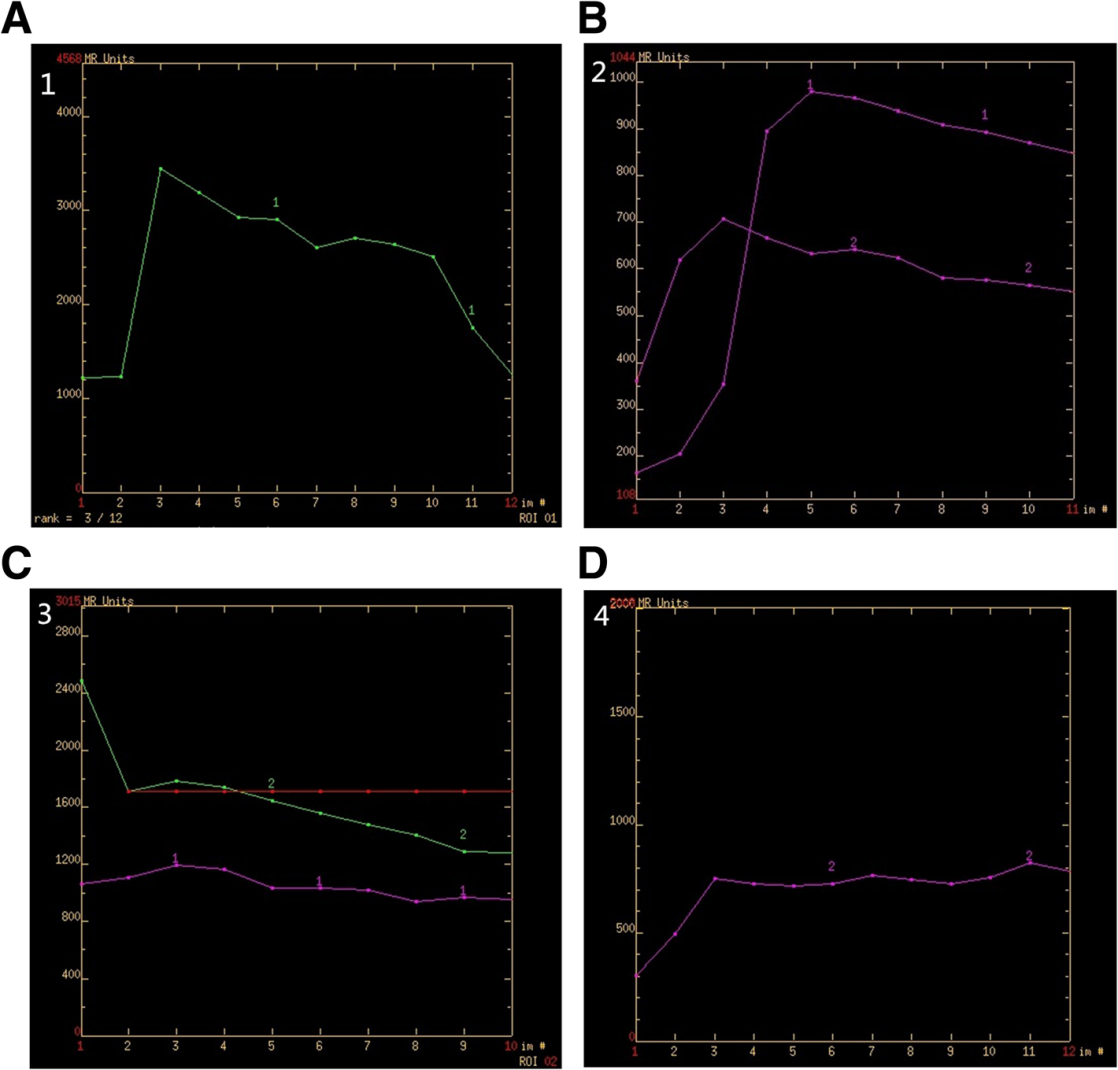
The enhancement curves of the lesions. **a**: Male, 59 years old, High-differentiation HCC with rapid increase and rapid Decrease; **b**: Female, 40 years old, Hemangioma (Curve 1 is the enhancement curve of the lesion with rapid increase and slow decrease, and Curve 2 is the enhancement curve of normal liver tissue); **c**: Male, 51 years old, HCC (Curve 1 is the enhancement curve of the lesion with a slow increase and slow decrease, and Curve 2 is the enhancement curve of the abdominal aorta); **d**: Male, 63 years old, Cholangiocarcinoma (The enhancement curve of the lesion has a slow increase after no apparent decline)

After data acquisition, all of the images were transferred to the AW4.3 workstation and analyzed by Functool analysis software. ROI was drawn in the maximum cross-section of the lesion in a relatively uniform signal intensity excluding the vessels, hemorrhage, necrosis, and cystic degeneration. Each measurement of the ROI was repeated by 3 times for statistical analysis. The Mean enhancement time (MET), positive enhancement integral (PEI), a maximum slope of increase (MSI), and a maximum slope of decrease (MSD) values and the curve types of ROIs were calculated and shown by Functool analysis software. The curve types of ROIs were divided into the following 4 categories: I, increase rapidly and decrease rapidly; II, increase rapidly and decrease slowly; III: increase slowly and decrease slowly; and IV, rise slowly after no apparent decline (Fig. 2a-d).

### Data analysis

The statistical software package SPSS 20.0 (SPSS Inc., Chicago, IL, USA) was used for data analysis. We performed normal testing and homogeneity of variance testing for MET, PEI, MSI, and MSD values in the benign and malignant tumor groups, such as hemangioma, HCC, cholangiocarcinoma, and metastatic tumor groups. The Student *t*-test was used for comparison of each parameter value between benign and malignant hepatic tumors. Nonparametric analyses including the Kruskal-Wallis test and Wilcoxon-Mann-Whitney U test using the Bonferroni correction were performed on data of parameter values among four groups, and Fisher exact probability test was used in the analysis of the curve types among the four groups. Receiver Operator Characteristic (ROC) curves were evaluated to compare every parameter value in the differential diagnoses of hepatic tumors. The area under the curve (AUC) reflects the accuracy of diagnostic testing, in which the value of AUC in 50 ~ 70, 70% ~ 90%, and > 90% corresponding to low, medium, and higher the accuracy of diagnostic testing respectively. Logistic regression analysis was used to obtain the regression eq. A *P* value < 0.05 was considered statistically significant.

## Results

### Comparison of benign and malignant hepatic tumors

The MET, PEI, and MSI values were significantly lower in the malignant hepatic tumor group than that in the benign tumor group (Table 1). Different lesions had different curve types, with benign tumors presenting with type II and malignant tumors mainly presenting with type I (Table 2).

**Table 1:** Comparison of each parameter value between benign and malignant hepatic tumors [M(QL~Qu)]

Group	MET*	PEI	MSI	MSD
Benign tumor	596.06 (516.01~596.06)	256.95 (36.50~421.59)	283.47 (162.12~437.08)	88.09 (77.13~153.69)
Malignant tumor	506.94 (478.48~591.06)	32.24 (3.34~99.07)	91.13 (50.95~150.53)	121.09 (80.94~161.74)
*T* value	2.53	2.77	3.76	1.02
*P* value	0.012	0.006	0.001	0.309

Remarks: Wilcoxon test,* Unit: s

**Table 2:** Comparison of the curve types between benign and malignant tumors (case)

Group	Curve types			
	I	II	III	IV
Benign tumor	0	10	0	3
Malignant tumor	31	9	5	22
*P* value	< 0.001			

Remark: Fisher exact probability test

### Comparison between malignant hepatic tumors

**Fig. 3:**
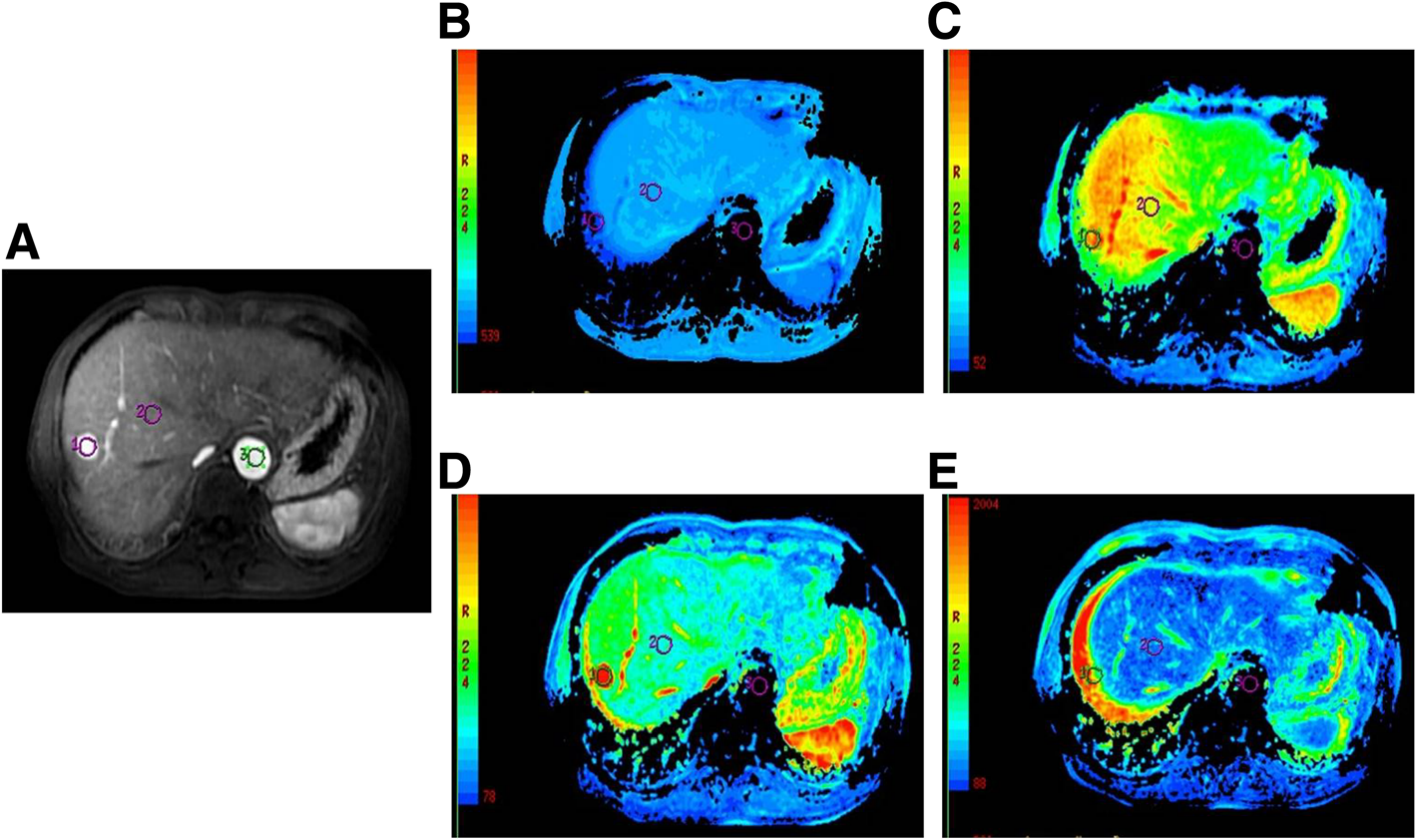
Imaging from a patient of male, 59 years old with high differentiation HCC. Figs. **a**-**e** represent dynamic contrast-enhanced magnetic resonance imaging and the pseudo-color images of MET, PET, MSI, and MSD, respectively

The PEI, MSI and MSD values among hemangiomas, HCCs, cholangiocarcinomas, and metastatic tumors had significant differences (Fig. 3a-e and Table 3). HCCs mainly presented with type I, metastatic tumors, and cholangiocarcinomas mainly presented with type IV, and hemangiomas mainly presented with type II (Table 4).

**Table 3:** Comparison of each parameter value among four groups [M(QL~Qu)]

Group	MET*	PEI	MSI	MSD
HCC	528.14 (476.25~592.40)	20.51 (2.94~83.35)	83.93 (49.51~133.41)	131.65 (90.51~182.63)
Metastatic tumor	507.59 (505.30~534.64)	52.91 (31.89~114.30)	84.76 (49.37~130.52)	44.76 (42.49~57.24)
Cholangiocarcinoma	502.84 (491.57~546.42)	97.35 (47.92~183.20)	166.13 (128.10~233.93)	129.57 (105.80~147.67)
Hemangioma	536.35 (505.30~596.06)	353.89 (133.10~517.43)	340.72 (265.70~633.86)	88.09 (80.84~217.85)
*F* value	0.99	14.69	24.40	15.92
*P* value	0.803	0.002	< 0.001	0.001

Remark: Kruskal-Wallis test

**Table 4:** Comparison of the curve types among the four groups (case)

Group	Curve types			
	I	II	III	IV
HCC	31	7	4	13
Metastatic tumor	0	1	1	4
Cholangiocarcinoma	0	1	0	5
Hemangioma	0	10	0	1
*P* value	< 0.001			

Remark: Fisher exact probability test

### Diagnosis of malignant hepatic tumor

**Fig. 4:**
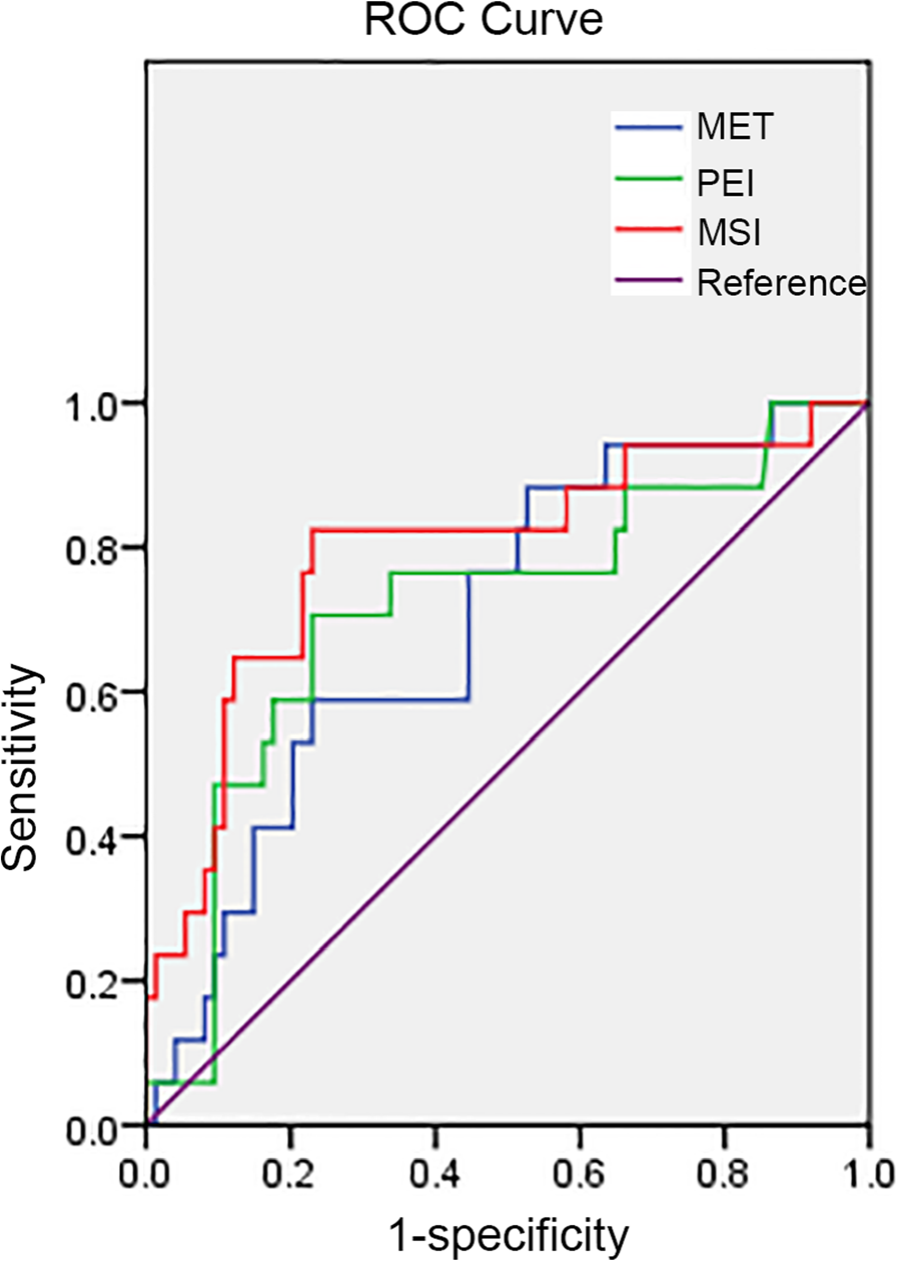
ROC curves for MET, PEI, and MSI. Abbreviations: MET: Mean enhancement time; PEI: positive enhancement integral; MSI: a maximum slope of increase

The AUC values of the ROC curves for MET, PEI, and MSI were 0.70, 0.72, and 0.80 respectively, all of which had diagnostic significances (Fig. 4). The specificity of MET, PEI, and MSI were all 77%, and the sensitivity was 58.80, 70.60, and 82.40% retrospectively (Table 5). Logistic regression analysis showed the regression equation to be *P* = 1/[1 + e^0.008 × 1 + 0.007 × 2–6.707^], and taking the Youden index (sensitivity + specificity − 1) maximum points as the diagnostic point was 0.2946. When the regression equation calculated value was less than the diagnostic point, it is more likely to be a malignant tumor (Table 6).

**Table 5:** ROC curve analysis of each parameter

Parameter	AUC	Standard error	*P* value	95% Confidence interval	Threshold value	Sensitivity (%)	Specificity (%)
MET	0.70	0.07	0.01	0.57~0.83	592.13	58.8	77
PEI	0.72	0.07	0.01	0.57~0.86	120.11	70.6	77
MSI	0.8	0.07	0.00	0.66~0.92	156.97	82.4	77

Remark: Fisher exact probability test

**Table 6:** Results of logistic regression analysis

Covariant	Regression coefficient	Standard error	*P* value
X1	0.008	0.003	0.011
X2	0.007	0.002	0
X3	0	0.001	0.543
constant	−6.707	1.892	0

Remark: X1 present with MET, X2 present with MSI, X3 present with PEI

## Discussion

In this present study, we found that MDCE-MRI showed different characteristics of enhancement by a time-signal intensity curve, the various parameters in assessing the degree of tumor neovascularization non-invasively. These parameters can be used for the quantitative analysis of diseases. The MET reflects the average pass time, the PEI relates to the relative blood volume, the MSI represents the microcirculatory blood flow, and the MSD reflects the blood supply of the tumor velocity [9, 10]. The mean values of MET, PEI, and MSI for benign tumors were higher than malignant tumors, and the difference was statistically significant. When the thresholds were 592.13, 120.11, and 156.97 respectively, the diagnostic specificity of these parameters were 77%, and the sensitivities were 58.80, 70.60, and 82.40% respectively. A large proportion of benign tumors is hemangiomas and the pathology of hemangiomas is mainly composed of abnormal dilatation of hepatic sinusoids. Although the MSD of benign tumors was less than malignant tumors, the difference was not statistically significant. Based on the literature, many factors can affect the enhancement type of the tumor, such as the density and permeability of the tumor vessels and extracellular diffusion space [11, 12]. We compared each parameter value among the four groups of tumors and found that the PEI and MSI values between hemangiomas, HCCs, cholangiocarcinomas, and metastatic tumors had significant differences. The MSD values between metastatic tumors and HCCs also had significant differences. Furthermore, the MET, PEI, and MSI values of hemangiomas were all higher than other tumors.

These results may have a relationship to the pathology of hemangiomas, HCCs, cholangiocarcinomas, and metastatic tumors [13–16]. The pathology of hemangiomas is composed of abnormal dilatation of hepatic sinusoids; however, HCCs develop in multistage processes with an increase in the degree of malignancy as the greater the blood supply in the hepatic artery. Cholangiocarcinomas are hypovascular tumors with increased fiber composition. According to the varied origin of cholangiocarcinomas, the blood supply of metastatic tumors is different and the rate of growth is usually rapid, thus the center of the tumor is prone to be liquefaction necrosis. Therefore, blood flow in hemangiomas is higher than other liver diseases.

Some research have suggested that parameters which are obtained from MDCE-MRI are highly related to tumor angiogenesis [17]. Because different lesions have different hemodynamic metabolic characteristics, the enhancement curve types were varied, which reflects the blood supply of tissues and vascular permeability state. Combined with the enhancement type curve of this study, we divided the curve type into the four categories. We found that there was a significant difference between benign and malignant tumors, as well as among each four groups. Benign tumors present with type II and malignant tumors mainly present with type I. HCCs mainly present with type I, metastatic tumors and cholangiocarcinomas mainly present with type IV, and hemangiomas mainly present with type II, all of which are consistent with reports in the literature [18, 19]. This result reflects the pathologic changes of each lesion. The histologic characteristics of HCCs reflect the liver arterial blood supply, the histologic characteristics of cholangiocarcinomas reflect the hypovascular tumor containing more fibers, which results in slow contrast agent diffusion between the blood vessels and fibrous tissues. The pathologic features of hemangiomas reflect abnormal dilatation of hepatic sinusoids and the enhanced feature of metastasis depends on the blood supply of the primary tumor.

In addition, we adopted parameters that were significantly different between benign and malignant tumors and performed logistic regression analysis. We obtained the regression eq. (*P* = 1/[1 + e^0.008 × 1 + 0.007 × 2–6.707^]) and took the Youden index maximum points as the diagnostic point, which was 0.2946. When the regression equation calculated value was less than the diagnostic point, the tumor was malignant.

Due to the small sample size, there are several limitations in the article, such as, it is not possible to characterise all metastases into a single group. There is no discussion of dysplastic nodules versus HCC. And HCCs accounted for a larger proportion of the tumors in the current study. For benign tumors, hemangiomas accounted for a larger proportion. There is no mention of differences between cirrhotic and normal background livers. The main focus of the discussion is on haemangiomas, which are almost always easy to diagnose. We will increase sample size for further research.

## Conclusion

Parameters obtained from MDCE-MRI can reflect the hemodynamic characteristics of lesions, which has application value in differential diagnoses of hepatic masses. A larger study is needed to validate the findings.

## Abbreviations

AFP: alpha-fetoprotein
Gd-DTPA: Gadolinium-diethylene triamine pentaacetic acid
HCCs: hemangiomas, hepatocellular carcinomas
LAVA: Liver acceleration volume acquisition
MDCE-MRI: multiphase dynamic contrast-enhanced MRI
MRI: magnetic resonance imaging

## References

[1] Zhou X, Luo Y, Peng YL, Cai W, Lu Q, Lin L, Sha XX, Li YZ, Zhu M (2011). Hepatic perfusion disorder associated with focal liver lesions: contrast-enhanced US patterns--correlation study with contrast-enhanced CT. Radiology.

[2] Song P, Feng X, Inagaki Y, Song T, Zhang K, Wang Z, Zheng S, Ma K, Li Q, Kong D (2014). Clinical utility of simultaneous measurement of alpha-fetoprotein and des-gamma-carboxy prothrombin for diagnosis of patients with hepatocellular carcinoma in China: a multi-center case-controlled study of 1,153 subjects. Bioscience trends.

[3] McGlynn KA, London WT (2005). Epidemiology and natural history of hepatocellular carcinoma. Best Pract Res Clin Gastroenterol.

[4] Lee VS, Lavelle MT, Rofsky NM, Laub G, Thomasson DM, Krinsky GA, Weinreb JC (2000). Hepatic MR imaging with a dynamic contrast-enhanced isotropic volumetric interpolated breath-hold examination: feasibility, reproducibility, and technical quality. Radiology.

[5] Semelka RC, Shoenut JP, Kroeker MA, Greenberg HM, Simm FC, Minuk GY, Kroeker RM, Micflikier AB. Focal liver disease: comparison of dynamic contrast-enhanced CT and T2-weighted fat-suppressed, FLASH, and dynamic gadolinium-enhanced MR imaging at 1.5 T. Radiology 1992;184: 687–694. 10.1148/radiology.184.3.1324509 1324509

[6] Isozaki T, Numata K, Kiba T, Hara K, Morimoto M, Sakaguchi T, Sekihara H, Kubota T, Shimada H, Morizane T (2003). Differential diagnosis of hepatic tumors by using contrast enhancement patterns at US. Radiology.

[7] Kim MJ, Kim JH, Chung JJ, Park MS, Lim JS, Oh YT (2003). Focal hepatic lesions: detection and characterization with combination gadolinium- and superparamagnetic iron oxide-enhanced MR imaging. Radiology.

[8] Mostardi PM, Glockner JF, Young PM, Riederer SJ (2011). Contrast-enhanced MR angiography of the abdomen with highly accelerated acquisition techniques. Radiology.

[9] Kudo M, Tomita S, Tochio H, Kashida H, Hirasa M, Todo A (1991). Hepatic focal nodular hyperplasia: specific findings at dynamic contrast-enhanced US with carbon dioxide microbubbles. Radiology.

[10] Masselli G, Picarelli A, Di Tola M, Libanori V, Donato G, Polettini E, Piermattei A, Palumbo P, Pittalis A, Saponara A (2010). Celiac disease: evaluation with dynamic contrast-enhanced MR imaging. Radiology.

[11] Ito K, Fujita T, Shimizu A, Koike S, Sasaki K, Matsunaga N, Hibino S, Yuhara M (2004). Multiarterial phase dynamic MRI of small early enhancing hepatic lesions in cirrhosis or chronic hepatitis: differentiating between hypervascular hepatocellular carcinomas and pseudolesions. AJR Am J Roentgenol.

[12] Sun HY, Lee JM, Shin CI, Lee DH, Moon SK, Kim KW, Han JK, Choi BI (2010). Gadoxetic acid-enhanced magnetic resonance imaging for differentiating small hepatocellular carcinomas (< or =2 cm in diameter) from arterial enhancing pseudolesions: special emphasis on hepatobiliary phase imaging. Investig Radiol.

[13] Glazer GM, Aisen AM, Francis IR, Gyves JW, Lande I, Adler DD (1985). Hepatic cavernous hemangioma: magnetic resonance imaging. Work in progress Radiology.

[14] Koh TS, Thng CH, Lee PS, Hartono S, Rumpel H, Goh BC, Bisdas S (2008). Hepatic metastases: in vivo assessment of perfusion parameters at dynamic contrast-enhanced MR imaging with dual-input two-compartment tracer kinetics model. Radiology.

[15] Jeong MG, Yu JS, Kim KW (2000). Hepatic cavernous hemangioma: temporal peritumoral enhancement during multiphase dynamic MR imaging. Radiology.

[16] Shimizu A, Ito K, Koike S, Fujita T, Shimizu K, Matsunaga N (2003). Cirrhosis or chronic hepatitis: evaluation of small (<or=2-cm) early-enhancing hepatic lesions with serial contrast-enhanced dynamic MR imaging. Radiology.

[17] Elsayes KM, Narra VR, Yin Y, Mukundan G, Lammle M, Brown JJ (2005). Focal hepatic lesions: diagnostic value of enhancement pattern approach with contrast-enhanced 3D gradient-echo MR imaging. Radiographics.

[18] Lutz AM, Willmann JK, Goepfert K, Marincek B, Weishaupt D (2005). Hepatocellular carcinoma in cirrhosis: enhancement patterns at dynamic gadolinium- and superparamagnetic iron oxide-enhanced T1-weighted MR imaging. Radiology.

[19] Burns PN, Wilson SR (2007). Focal liver masses: enhancement patterns on contrast-enhanced images--concordance of US scans with CT scans and MR images. Radiology.

